# Serum estradiol level on the day of trigger as a predictor of number of metaphase II oocytes from IVF antagonist cycles and subsequent impact on pregnancy rates

**DOI:** 10.5935/1518-0557.20210007

**Published:** 2021

**Authors:** Héctor Salvador Godoy Morales, Miguel Loyo Guiot, Germán Gabriel Palacios López, Daniel Vieyra Córtes, Berenice Flores Maldonado, Hilda Sánchez Hernández, Griselda Claribel Reyes Torres, Francisco Miguel Rojas Camacho, Gabriela Ayala Montoya

**Affiliations:** 1 Angeles Pedregal Hospital, Assisted Reproduction Unit, Mexico City, Mexico

**Keywords:** estradiol, oocyte retrieval, trigger day, antagonists, pregnancy rate

## Abstract

**Objective:**

This study aimed to examine the association between serum estradiol levels and number of metaphase II oocytes harvested after in vitro fertilization cycles used in embryo transfers and the subsequent impact on pregnancy rates.

**Methods:**

This observational analytical retrospective study was carried out in 2010-2018 at the Angeles del Pedregal Hospital. It included 181 cases and looked into the number of metaphase II oocytes to predict pregnancy rates. Statistical analysis was based on the calculation of correlations between variables and logistic regressions.

**Results:**

Estradiol levels increased with the number of oocytes by a median correlation (r=0.482, p=0.000). On the day of trigger, estradiol levels predicted the number of retrieved oocytes with 23% reliability (R2=0.232, *p*=0.000); a linear trend correlation of r=0.489, *p*=0.000 was found between estradiol levels on the day of trigger and number of metaphase II oocytes.

**Conclusions:**

Serum estradiol on the day of trigger as a predictor of metaphase II oocytes in antagonist cycles encourages greater oocyte maturity and fertilization, whereas, in isolation, it does not determine the pregnancy achievement.

## INTRODUCTION

The main objective of controlled ovarian stimulation (COS) in in vitro fertilization (IVF) cycles is to produce a large number of mature oocytes. On the other hand, elevated secretion of ovarian steroid hormones is strongly associated with COS, and in this regard, serum estradiol (E_2_) levels may increase more than 10 times compared to levels found during spontaneous cycles. Serum estradiol levels have been used as a predictor at the time of oocyte retrieval (Macklon *et al*., 2006; Pittaway & Wentz, 1983).

Estradiol is the most potent estrogen (17 β-estradiol). It is synthesized by ovarian granulosa cells from androstenedione and testosterone, as the main product of ovary endocrine secretion (Wu *et al*., 2007; Brown, 1978). In the natural ovulatory cycle, estradiol concentrations range from 200 to 400 pg/mL just before luteinizing hormone (LH) levels peak. In exogenously stimulated cycles, similar concentrations are expected per follicle. However, the levels reported in the literature are very dissimilar (Speroff & Fritz, 2007). Additional studies based on cycles with antagonist protocols should be performed to establish more reliable parameters and reflect more accurately their effects in the Mexican population.

## MATERIALS AND METHODS

### Patient Characteristics

This observational descriptive cross-sectional retrospective study included the records of patients seen at the Fertility Clinic of the Angeles del Pedregal Hospital in Mexico City, from 2010 to 2018. The study included patients submitted to IVF diagnosed with infertility of any kind (primary or secondary) aged 18-56 years. The antagonist protocol was started when the patients reached a follicle size of 14 mm. Additional information such as serum estradiol levels prior to the trigger and cycle outcome, number of retrieved oocytes, number of metaphase II (MII) oocytes, and pregnancy rate were also captured. Patients achieving pregnancy with donor eggs or with associated male factor infertility and patients with exogenous estradiol supplementation were excluded. The social and clinical characteristics of the study group are shown in Table 1.

**Table 1:** Clinical characteristics of the study group

	Median	Minimum	Maximum
**Age (years)**	35.9	18	56
**Body mass index (kg/m^2^)**	18.5	36	24.5
**Height (m)**	1.62	1.57	1.77
**Baseline E2 (pg/ml)**	54.15	5	278
**Baseline anti-Müllerian hormone (ng/ml)**	1.95±2.08	0.003	8.11
**Baseline FSH (UI/ml)**	8.56	0.20	109
**Estradiol average level on the day of trigger per metaphase II oocyte (pg/ml)**	381	293.2	468.8
**Estradiol by age group (pg/ml)** **<35 years** **36-39 years** **>40 years**	304.01 349 273	41.36 54.5 40.16	1086 1118 1375

### Stimulation Protocol

Since infertility is a multifactorial condition, this study considered the most common diseases - including polycystic ovary syndrome (PCOS), fibroids, polyps and hypothyroidism - to decrease information bias. Patients started stimulation on day 2 with recombinant gonadotropin in most cases or an antagonist protocol with Cetrotide 0.25 mg, (when the follicle reached 14 mm), although some used highly purified urinary gonadotropin (Gonal F 150-300 IU/Merional 225-300 IU) in their cycles. Doses were established based on patient baseline characteristics (age, weight, FSH, anti-Müllerian hormone and antral follicle count) and were adjusted from the sixth day of stimulation based on ultrasound controls. Choriomon 10,000 IU was used in final oocyte maturation in all cases.

### *In Vitro* Fertilization and Embryo Transfer

Oocyte maturation was performed 36 hours prior to follicular puncture using urinary HCG; in patients at risk of ovarian hyperstimulation, a GnRH analogue was used at a dosage of 0.2 mg. In all cases, the Lübeck or antagonist protocol was used to prevent spontaneous ovulation. GnRH antagonists (Cetrorelix) were used at a dosage of 0.25 mg when a follicle greater than 14 mm was observed.

### Hormonal Determination

Serum estradiol measurements were performed in a single laboratory, belonging to the Angeles del Pedregal Hospital, since the samples were taken during the last appointment and sometimes on the day of retrieval. It is important to mention that the same medical team performed oocyte retrievals and estradiol level measurements.

### Statistical Analysis

Patients were selected based on non-probability sampling and the study method was non-participant observation. Data was collected from 181 records of female patients with infertility aged 18-56 years seen at the Fertility Clinic of the Angeles del Pedregal Hospital in Mexico City from 2010 to 2018 and recorded in data collection cards. No patients were lost or excluded from the study, since it included everyone whose records contained the measurements analyzed in the study.

Patients were divided into three age-based groups: Group I: patients aged ≤ 35 years; Group II: patients aged 36 and < 40 years; Group III: patients aged ≥ 40 years. The data sets analyzed in the study were captured from patient medical records and recorded in data collection cards.

The data from the medical records were reviewed and submitted to statistical analyses (Microsoft Excel^©^ and IBM SPSS Statistics^©^, Version 20). Inferential statistics was used to derive central tendency and dispersion measures for quantitative variables and proportions for qualitative variables. Pearson’s correlation coefficient was used to analyze the correlations between quantitative variables following a normal distribution. Spearman’s rank correlation coefficient was used to analyze variables not following a normal distribution, as well as the mean comparison tests for ANOVA independent variables of a pathway for normal distribution quantitative variables, and the determination coefficient obtained by logistic regression for predictor variables.

The Research Committee of the Angeles del Pedregal Hospital approved this study, which strictly adhered to the current guidelines of the General Health Law, Chapter I of the Ethical Aspects of Human Beings Research, Article 17.

## RESULTS

The medical records of 181 patients meeting the following inclusion criteria were analyzed: mean age of 35.9±6.19 years woman (18-56); baseline E_2_ of 54.15± 53.63 pg/ml (5-278); baseline anti-Müllerian hormone of 1.95±2.08 ng/ml (.003-8.11); baseline FSH of 8.56±12.76 IU/ml (0.20-109). The average estradiol level on the day of trigger was 217.8±217.8 pg/ml per retrieved oocyte and 381±87.8 pg/ml per metaphase II oocyte.

In terms of patient age, the following mean E_2_ levels were observed in each age group: 304.01±208.1 pg/ml (41.36-1086) in patients aged ≤ 35 years; 349±217.01 pg/ml (54.5-1118) in patients aged 36 to 39 years; and 273±221.75 pg/ml (40.16-1375) in patients aged 40 and over. Mean E_2_ levels were statistically different between groups (ANOVA 3.94, gl 2, *p*=0.021).

The correlation between serum estradiol levels and number of retrieved oocytes and MII oocytes was assessed. In general terms, the analysis of E_2_ levels and number of retrieved oocytes found that when E2 levels increased, the number of retrieved oocytes also increased. This correlation is of average strength and significant (r=0.482, *p*=0.000), particularly in the study groups in which the number of oocytes retrieved depended on E_2_ levels at a constant ratio. We found a positive correlation between number of oocytes and estradiol levels in the same age group, meaning that higher levels of estradiol and larger numbers of retrieved oocytes (r=0.482, *p*=0.000) presented a correlation of average strength. Correlations are deemed strong when r>.75.

**Figure 1:**
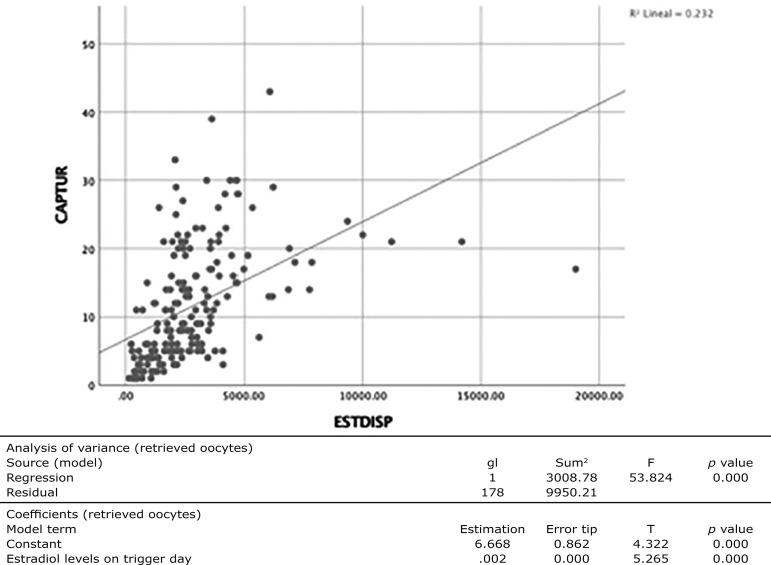
Analysis between the number of retrieved oocytes and E2 levels on the day of trigger.

Logistic regression analysis found a goodness-of-fit between E_2_ levels on the day of trigger and number of retrieved oocytes of 23% (R2=0.232, *p*=0.000), as shown in Figure 1.

**Figure 2:**
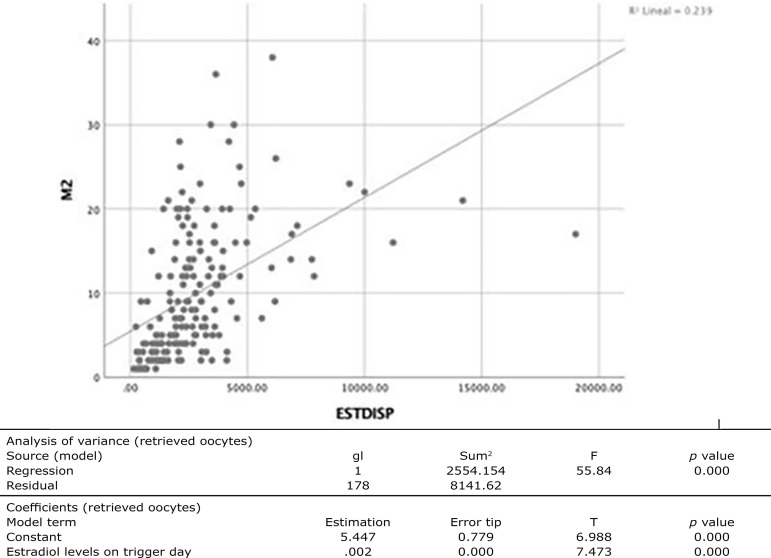
Analysis between the number of MII oocytes and E2 levels on the day of trigger.

Correlation analysis identified an association of average strength (r=0.489, *p*=0.000) between E_2_ levels on the day of trigger and number of MII oocytes. Logistic regression analysis found a goodness-of-fit of 23% (R^2^=0.239), as shown in Figure 2.

### Assessment of Estradiol Levels by Groups

After analyzing the general findings discussed above, we decided to analyze three different groups based on their respective serum E_2_ levels, as follows: Group 1 with levels up to 1000 pg/ml; Group 2: E_2_ levels from 1001 pg/ml to 3000 pg/ml; and Group 3 with levels >3001 pg/ml. The mean ages of the patients in each of the groups (36.7, 35, and 37.05 years) were not statistically different (*p*=0.106), as also seen in baseline E_2_ (*p*=0.358) and FSH (*p*=0.362) levels and number of follicles by the end of the stimulation (*p*=0.179).

Nevertheless, there were noticeable differences (*p*=0.000) in the number of retrieved oocytes in Group 3, in which the mean number of retrieved oocytes was 16.51±8.28 and MII oocytes was 14.16±7.49, thus confirming again that the higher the E_2_ levels the greater the number of oocytes obtained, with impacts seen also on higher rates of oocyte maturity and fertilization (*p*=0.000), as shown in Table 2.

**Table 2:** Main findings according to estradiol levels in three study groups with different E2 levels

Variable	Group 1 (E_2_ up to 1000 pg/ml)	Group 2 (E_2_ of 1001-3000 pg/ml)	Group 3 (E_2_ higher than 3000 pg/ml)	*p* value
Age (years)	36.7±5.93 (19-46)	35±6.51 (18-56)	37.05±5.67 (18-47)	0.106
Baseline estradiol (pg/ml)	49.5±37.25 (5-139)	61.63±69.58 (9-278)	44.95±22.08 (9-104)	0.358
Baseline FSH (IU/ml)	5.25±3.36 (0.2-14.7)	9.97±5.24 (1.4-10.9)	8.45±12 (2.7-70.6)	0.362
Follicles at the end of stimulation (n)	12.19±9.27 (0-34)	13.35±9.76 (1-48)	9.7±4.81 (1-19)	0.179
Retrieved oocytes (n)	3.77±3.32 (1-15)	10.65±7.28 (1-33)	16.51±8.28 (2-38)	0.000
Retrieved MII oocytes (n)	3.1±3.11 (1-15)	9.24±6.54 (1-28)	14.16±6.54 (2-38)	0.000
Oocyte maturity (%)	82.2	88	85	0.000
Fertilized (n)	2.35±2.65 (0-13)	6.48±5.61 (0-23)	10.52±7.49 (0-30)	0.000
Fertilization rate (%)	75.8	70.12	74	0.000

### Association between Estradiol and Pregnancy by Group

Regarding the association between E_2_ and pregnancy, the evaluation of E_2_ levels on the day of trigger did not reveal statistically significant results (*p*=0.681), which means that E_2_ levels were not associated with achieving pregnancy. This was further corroborated as parents were sorted into groups in terms of E_2_ levels as in the previous section: In group 1, patients with E_2_ levels up to 1000 pg/ml had a pregnancy rate of 32%; in group 2 with E_2_ levels from 1001 pg/ml to 3000 pg/ml, the pregnancy rate was 16%; and in group 3 with E_2_ levels > 3001 pg/ml, the pregnancy rate was 22.6%. Comparisons between groups for pregnancy, miscarriage, and live birth rates did not find statistically significant differences (*p*>0.05), as shown in Table 3. Therefore, serum E_2_ levels alone are not useful in estimating which patients will achieve pregnancy.

**Table 3:** Main findings according to estradiol levels in three study groups and their different reproductive results

Variable	Group 1 (E_2_ up to 1000 pg/ml)	Group 2 (E_2_ of 1001-3000 pg/ml)	Group 3 (E_2_ higher than 3000 pg/ml)	Kruskal- Wallis H	*p* value
Pregnancy rate (%)	32	34.8	32.8	0.102	0.950
Miscarriage rate (%)	16	13.5	11.5	0.083	0.959
Live birth rate (%)	22.6	11.5	21.3	0.435	0.804

## DISCUSSION

Controlled ovarian stimulation (COS) is a crucial step in current assisted reproductive technologies to obtain mature follicular cohorts. This is inevitably associated with supraphysiological levels of estradiol. Measuring serum E_2_ levels is part of the basic protocol in almost every assisted reproduction center in the world; in addition to predicting the number of expected oocytes, it is also useful in preventing inherent complications from stimulation cycles.

Establishing a more specific serum E_2_ value as a prognostic marker at the time of controlled ovarian stimulation provides for a more accurate picture of the expected outcomes.

The results seen in this study showed that E_2_ levels on the day of trigger predicted the number of retrieved oocytes with 23% reliability (R^2^=0.232, *p*=0.000), and that the number of oocytes to be retrieved on average is linked to a value of 300 pg/ml in antagonist protocol cycles, unlike classic studies, which reported very wide ranges of estradiol levels (200-400 pg/ml per oocyte) (Speroff & Fritz, 2007).

After consulting the available global literature, no reports were found correlating such accurate pre-trigger estradiol levels and number of retrieved oocytes and MII oocytes obtained from ovarian stimulation cycles using antagonists (Agard *et al*., 2007).

Secondary analysis of the estradiol levels of patients who achieved pregnancy after ovarian stimulation cycles found no significant difference or different values when comparing their results against the patients who did not become pregnant. In group 1, the pregnancy rate was 32%; in group 2 it was 34.8%; and in group 3 it was 32.8%. The pregnancy, miscarriage, and live birth rates of the groups were not statistically different (*p*>0.05). The latter findings agree with a study in which serum estradiol, progesterone and B-HCG levels were analyzed; the authors found that elevated estradiol levels were not statistically associated with achieving pregnancy (Carmona *et al*., 2003); another study, however, reported that high E_2_ levels were correlated with pregnancy (Blazar *et al*., 2004) and low E_2_ levels were associated with decreased pregnancy rates (Kondapalli *et al*., 2012).

Controversy is present in studies, with reports of associations between estradiol to mature oocyte ratios greater than 400 pg/ml/oocyte in normal responders yielding lower clinical pregnancy (CPR), ongoing pregnancy (OPR) and live birth (LBR) rates than individuals treated in regimens with < 200 pg/ml/oocyte and 200-300 pg/ml/oocyte (CPR 1% *vs*. 16% and 32%, respectively, *p*=0.017; OPR 0 *vs*. 15% and 27%, respectively, *p*=0.011; and LBR 0 *vs*. 13% and 26%, respectively, *p*=0.018) (Sandoval *et al*., 2016).

Previous studies reported that the ideal E_2_ range to achieve pregnancy in assisted reproduction cycles is between 1000 to 3148 pg/mL (Sandoval *et al*., 2016; Kondapalli *et al*., 2012). This range is broad and unspecific, and shows that the use of this parameter may not be reliable, like many other hormonal markers that have been used to predict pregnancy rates, such as the AMH (Tal *et al*., 2015). In our study, the ideal estradiol level to achieve pregnancy was not analyzed, although group 2 had a pregnancy rate 34.8% higher than the rates seen in groups 1 and 2 (*p*=0.950).

It is worth mentioning that low pregnancy rates may be associated with estradiol levels, an essential element in endometrial and placental development. Simón *et al*. (1995) demonstrated that elevated estradiol levels negatively affected endometrial receptivity and that elevated levels in fresh cycles before transfer correlated with worse implantation outcomes. Farhi *et al*. (2010) reported that elevated E_2_ levels during IVF cycles were linked to a higher incidence of adverse pregnancy outcomes.

Future studies should assess whether patients prescribed GnRH analog trigger due to high estradiol levels might suffer from adverse effects other than the risk of ovarian hyperstimulation such as increased miscarriage rates and, paradoxically, lower pregnancy rates, as observed by some authors (Steward *et al*., 2015).

## CONCLUSIONS

Numerous studies hake looked into estradiol levels on the day of trigger and their impact on ovarian stimulation cycles. However, as mentioned above, few have been conducted to predict the association between estradiol levels, metaphase II oocytes and their correlation with pregnancy rates.

Estradiol is critical in endometrial and placental development; however, excess E_2_ in the early stages of pregnancy may have adverse effects on placentation. Elevated E_2_ has been shown to adversely affect endometrial receptivity; and high E_2_ levels in fresh cycles prior to transfer have been correlated with decreased implantation rates. Additionally, elevated E_2_ levels during IVF cycles have been linked to increased incidence of adverse pregnancy outcomes, including preeclampsia and intrauterine growth restriction (Hagerman & Villee, 1953; Simón et al., 1995; Farhi *et al*., 2010).

Serum estradiol levels of 308.6pg/ml/oocyte collected on the day of trigger and on the day of egg retrieval during high complexity assisted reproduction cycles with antagonist protocols have been correlated with the number of retrieved oocytes and MII oocytes. Therefore, increases or decreases in one parameter affect its relationship with other parameters regardless of age, although they have not been associated with achieving or predicting pregnancy (*p*>0.05).

The limitations of this study include its retrospective nature, which makes it impossible to strictly control the factors affecting estradiol levels. Fortunately, in this study all measurements were made in the same laboratory coordinated by the same medical team. The next steps in this scarcely researched topic might include collecting more patient data, conducting more extensive studies to look into each specific situation, and perform randomized and prospective studies.

Studies enrolling specific populations serve as the foundation for epidemiological studies developed to institute personalized patient care based on findings derived from patients with different genetic traits under diverse environmental conditions treated in different corners of the world. The development of individualized treatment protocols that harness the power of predictive tools is one of the goals of future medical practice and patient management.

## References

[r1] Agard J, Glujovsky D, Shamonki MI, Frattarelli J, Bergh PA (2007). Estradiol levels after human chorionic gonadotropin (hCG) administration are not predictive of IVF outcome: analysis of 7,474 initial fresh IVF cycles. Fertil Steril.

[r2] Blazar AS, Hogan JW, Frankfurter D, Hackett R, Keefe DL (2004). Serum estradiol positively predicts outcomes in patients undergoing in vitro fertilization. Fertil Steril.

[r3] Brown JB (1978). Pituitary control of ovarian function--concepts derived from gonadotrophin therapy. Aust N Z J Obstet Gynaecol.

[r4] Carmona F, Balasch J, Creus M, Fábregues F, Casamitjana R, Cívico S, Vidal E, Calafell JM, Moreno V, Vanrell JA (2003). Early hormonal markers of pregnancy outcome after in vitro fertilization and embryo transfer. J Assist Reprod Genet.

[r5] Farhi J, Ben-Haroush A, Andrawus N, Pinkas H, Sapir O, Fisch B, Ashkenazi J (2010). High serum oestradiol concentrations in IVF cycles increase the risk of pregnancy complications related to abnormal placentation. Reprod Biomed Online.

[r6] Hagerman DD, Villee CA (1953). Effects of estradiol on the metabolism of human endometrium in vitro. J Biol Chem.

[r7] Kondapalli LA, Molinaro TA, Sammel MD, Dokras A (2012). A decrease in serum estradiol levels after human chorionic gonadotrophin administration predicts significantly lower clinical pregnancy and live birth rates in in vitro fertilization cycles. Hum Reprod.

[r8] Macklon NS, Stouffer RL, Giudice LC, Fauser BC (2006). The science behind 25 years of ovarian stimulation for in vitro fertilization. Endocr Rev.

[r9] Pittaway DE, Wentz AC (1983). Evaluation of the exponential rise of serum estradiol concentrations in human menopausal gonadotropin-induced cycles. Fertil Steril.

[r10] Sandoval JS, Steward RG, Chen C, Li YJ, Price TM, Muasher SJ (2016). High Peak Estradiol/Mature Oocyte Ratio Predicts Lower Clinical Pregnancy, Ongoing Pregnancy, and Live Birth Rates in GnRH Antagonist Intracytoplasmic Sperm Injection Cycles. J Reprod Med.

[r11] Simón C, Cano F, Valbuena D, Remohí J, Pellicer A (1995). Clinical evidence for a detrimental effect on uterine receptivity of high serum oestradiol concentrations in high and normal responder patients. Hum Reprod.

[r12] Speroff L, Fritz MA (2007). Endocrinología ginecológica clínica y esterilidad.

[r13] Steward RG, Zhang CE, Shah AA, Yeh JS, Chen C, Li YJ, Price TM, Muasher SJ (2015). High Peak Estradiol Predicts Higher Miscarriage and Lower Live Birth Rates in High Responders Triggered with a GnRH Agonist in IVF/ICSI Cycles. J Reprod Med..

[r14] Tal R, Tal O, Seifer BJ, Seifer DB (2015). Antimüllerian hormone as predictor of implantation and clinical pregnancy after assisted conception: a systematic review and meta-analysis. Fertil Steril.

[r15] Wu CH, Kuo TC, Wu HH, Yeh GP, Tsai HD (2007). High serum estradiol levels are not detrimental to in vitro fertilization outcome. Taiwan J Obstet Gynecol.

